# Factors Influencing Right Hemisphere Engagement During Metaphor Comprehension

**DOI:** 10.3389/fpsyg.2018.00414

**Published:** 2018-03-28

**Authors:** Michele T. Diaz, Anna Eppes

**Affiliations:** Department of Psychology, Pennsylvania State University, University Park, PA, United States

**Keywords:** metaphor comprehension, fMRI neuroimaging, semantics, right hemisphere, figurative language

## Abstract

Although the left hemisphere is critical for language, clinical, behavioral, and neuroimaging research suggest that the right hemisphere also contributes to language comprehension. In particular, research has suggested that figurative language may be one type of language that preferentially engages right hemisphere regions. However, there is disagreement about whether these regions within the right hemisphere are sensitive to figurative language *per se* or to other factors that co-vary with figurativeness. In this article, we will review the neuroimaging literature on figurative language processing, focusing on metaphors, within the context of several theoretical perspectives that have been proposed about hemispheric function in language. Then we will examine three factors that may influence right hemisphere engagement: novelty, task difficulty, and context. We propose that factors that increase integration demands drive right hemisphere involvement in language processing, and that such recruitment is not limited to figurative language.

## Introduction

Figurative or non-literal language is an ever-present aspect of communication that contributes to the vividness, richness, and efficacy of language. Figurative language comes in many forms (metaphors, idioms, jokes, sarcasm) and broadly includes any text where the intended meaning varies from a direct literal translation. For example, a metaphor can provide a succinct, vivid, and richly connotative description by drawing parallels between two distinct concepts (e.g., Crime is a disease vs. Crime is a beast, Thibodeau and Boroditsky, 2011). Figurative language differs from literal text in that there are both literal and figurative meanings. Although, the manner in which these two meanings are accessed (serially or in parallel) has been debated (Grice, 1975; Searle, 1979; Glucksberg et al., 1982; Blasko and Connine, 1993; McElree and Nordlie, 1999; Stern, 2000; Glucksberg, 2003), and context may further influence how these items are processed. Beyond the aspect of dual meaning, figurative language often differs from literal text along several dimensions including valence, ease of integration, comprehensibility, novelty, and the amount of context that is provided. Indeed, one of the challenges in examining rich, naturalistic discourse is that many of these interesting and influential factors covary among themselves, and it is challenging to control for such factors. Thus, it is unclear whether differences in processing figurative language are due to figurativeness *per se* or to variation in other factors that covary with figurativeness. Additionally, these same factors can vary within figurative language itself. For example, conventional metaphors, also known as frozen, familiar, or dead metaphors, such as ‘He’s falling in love.’ may become lexicalized or embedded in semantics similar to a definition of a single word because of their frequent use. When this occurs, processing such metaphors may be quite similar to understanding literal sentences. On the other hand, understanding novel instances of figurative language requires the dynamic integration of several distinct concepts. For example, the metaphor “Summer was a thousand colors in a parched landscape.” (Lee, 1960) evokes images of vivid and varied wildlife in bloom during the heat of a season.

In this mini-review, we will consider several theoretical perspectives on brain lateralization and figurative language. Then we will discuss several factors that may influence the processing demands and subsequent hemispheric recruitment of figurative and literal language: novelty, task difficulty, and context effects.

## Theoretical Perspectives on Hemispheric Function in Language

The left hemisphere’s importance for language in the vast majority of individuals has been validated with behavioral and neuroimaging experiments in healthy young adults (e.g., Price, 2012; Kemmerer, 2015) and with neurological patient studies (Broca, 1861; Dejerine, 1891; Davis and Wada, 1978; Mirman et al., 2015). However, the role of the right hemisphere in language is debated and its involvement likely varies depending on the particular aspect of language one might consider. For example, we might agree that syntactic computations and language production are largely based in the left hemisphere, whereas early acoustic processes and semantic processes, particularly visual aspects of semantics have a more bilateral organization (e.g., Hickok and Poeppel, 2007). Much of our understanding about whether a given brain region is necessary for a given function comes from experiments with neurological patients (e.g., Mirman et al., 2015). With respect to figurative language, particularly semantic aspects of figurative language, several theoretical viewpoints have been proposed about right hemisphere function. The Graded Salience Hypothesis (GSH, Giora, 1997, 1999, 2003) proposes that hemisphere involvement in language comprehension is influenced by the salience of a text rather than by figurativeness *per se*. According to the GSH, an item’s salience, or prominence and ease of processing, is influenced by several factors including conventionality, the item’s familiarity, how often the item is encountered, and the amount of context that is provided. An item’s salience will then determine how easily the words or phrases are processed, whether alternative interpretations are activated, and which hemisphere(s) are recruited. Moreover, the framework hypothesizes that highly salient words and phrases are supported predominantly by the left hemisphere while less salient linguistic material is also supported by cognitive resources in the right hemisphere. For example, when encountering a highly familiar metaphor such as ‘The city was dead last night.’ The figurative meaning would have the highest salience, entail low processing demands, and recruit left hemisphere neural resources, with the literal meaning likely never even being considered.

An alternative framework, the Coarse Coding Hypothesis, was proposed by Beeman and colleagues (Chiarello, 1988; Beeman et al., 1994; Beeman and Chiarello, 1998; Jung-Beeman, 2005) and suggests that both hemispheres contribute to “semantic activation, integration, and selection” with the left hemisphere supporting more focal semantic activation, and the right hemisphere supporting wider, or coarser, semantic interpretations. In this context, novel figurative language that forges associations between disparate or unfamiliar semantic concepts would engage the right hemisphere, whereas literal language and conventional figurative language would be supported largely by the left hemisphere.

A series of electrophysiological studies by Federmeier and colleagues have proposed a slightly different conceptualization of left and right hemisphere function (Federmeier and Kutas, 1999a,b, 2002; Federmeier et al., 2002). Although their work focused on literal sentence processing, it sheds light on the right hemisphere’s contribution to semantic processing by considering semantic distance. In this series of studies, participants read sentences and the authors manipulated the expectancy and semantics of the sentence final word. The final word could be the most expected word or an unexpected word that was either closely or more distantly related to the most expected word (e.g., “They wanted to make the hotel look more like a tropical resort. So, along the driveway they planted rows of *palms/pines/tulips*.”). Interestingly, although pines and tulips were equally unexpected, they found a reduction in the N400 response (an electrophysiological index of semantic processing), to words from the same semantic category as the most expected item (palm), but only when those items were initially presented to the left hemisphere. This suggests that left hemisphere processing resources had generated a prediction (at least at the level of the category). In contrast, both unexpected items elicited equivalently larger N400 responses compared to the most expected item when initially presented to the right hemisphere (Federmeier, 2007). Taken together, these findings suggest that the left hemisphere uses context to predict upcoming items, whereas the right hemisphere, while sensitive to integration demands, integrates material as it is presented. Applying this framework to figurative language we would expect left hemisphere resources to be involved most strongly in highly predictive contexts (e.g., familiar metaphors) and the right hemisphere to be more involved with text that has high integration demands (e.g., novel figurative language).

Comparing these three theoretical frameworks about hemispheric function, each makes similar claims regarding when the left and right hemisphere would be recruited. However the conceptual frameworks differ in terms of the underlying mechanism (salience, coarse coding, integration).

## Neural Data Supporting a Hemispheric Distinction for Metaphor Comprehension

Much of our initial understanding about hemispheric contributions to figurative language processing originates from seminal work observing neurological patients with right hemisphere brain damage who had difficulty processing metaphors (McIntyre et al., 1976; Winner and Gardner, 1977; Bryan, 1988; Brownell et al., 1990; Rinaldi et al., 2004), sarcasm (Giora et al., 2000), idioms (Van Lancker and Kempler, 1987; Kempler et al., 1999), and jokes (Bihrle et al., 1986). But see (Tompkins, 1990; Gagnon et al., 2003) for an alternative account.

While patient work can provide insight into brain regions that are crucial for various cognitive functions, neuroimaging techniques can be used to provide insight into language function in neurologically intact younger and older adults (Huettel et al., 2014). Consistent with much of the patient research, fMRI investigations of written sentential metaphors have often found engagement of the right hemisphere (Bottini et al., 1994; Eviatar and Just, 2006; Stringaris et al., 2006; Ahrens et al., 2007; Chen et al., 2008; Schmidt and Seger, 2009; Yang et al., 2009). Similar engagement of the right hemisphere has been found for auditorily presented metaphors compared to literal sentences (Obert et al., 2014). Specifically, comparisons of literal and metaphoric sentences have found that metaphoric sentences engage right frontal (e.g., right insula, Schmidt and Seger, 2009) and IFG (Bottini et al., 1994; Stringaris et al., 2006; Ahrens et al., 2007; Schmidt and Seger, 2009) as well as right temporal cortices, inferior (Eviatar and Just, 2006; Ahrens et al., 2007) and middle (Bottini et al., 1994; Chen et al., 2008), to a greater extent than literal sentences. However, not all investigations have shown right hemisphere engagement when processing metaphors (Rapp et al., 2004, 2007; Lee and Dapretto, 2006; Mashal et al., 2009). This pattern of results returns to the issue of whether the right hemisphere is sensitive to metaphors *per se*, or whether the right hemisphere might be sensitive to other variables that covary with figurative language such as novelty, task difficulty, or context. Consistent with the theoretical frameworks outlined earlier, each of these factors is likely to influence an item’s salience, the broadness of the semantic processing required, and the overall integration demands. Here we focus on how three of these variables (novelty, task difficulty, and context) have been examined and how each has influenced hemispheric recruitment (see **Table 1**).

**Table 1:** Variables that influence hemispheric recruitment.

Factor	Definition	Example
Novelty	A relative rating of the uniqueness of an item.	The flowers were an oasis. A sailboat is a floating leaf.^+^
Comprehension Difficulty	Generally a subjective, relative rating of how difficult an item is to understand.	Respect is a precious gem. The waltz is the nightingale of dance.^∗^
Context	The linguistic surroundings of an item. This can range from a single word to an entire sentence or paragraph.	Babies – angels. Due to the bad weather, the flight was bumpy.


Examples taken from ^+^Diaz et al. (2011) and ^∗^Schmidt and Seger (2009).

## Novelty Effects in Figurative Language

As previously mentioned, novelty in figurative or literal text is one aspect of language that influences processing time. Novelty is generally assessed by having participants rate the novelty of a given sentence or word pair. It can also be manipulated through prototypicality assessments of particular words (e.g., a robin being more familiar or prototypical compared to an ostrich). Yang and colleagues manipulated novelty in sentential metaphors using valence and imagery tasks (Yang et al., 2009). During both tasks, novel metaphors engaged right inferior frontal gyrus to a greater extent compared with both familiar metaphors and literal sentences. Moreover, during the imagery task, novel metaphors engaged right inferior temporal gyrus more than literal text.

Work from our lab extended these findings by examining how novelty influences neural recruitment in both literal and figurative sentences (Diaz et al., 2011). We constructed a series of literal and metaphoric sentences that varied in novelty, as rated by an independent sample of adults. In literal sentences, novelty was explicitly manipulated by varying the prototypicality of semantic features, based on published norms (McRae et al., 1999, 2005). We found a graded influence of both novelty and figurativeness: all novel sentences, as well as familiar metaphors engaged right inferior frontal gyrus and right temporal pole more than familiar literal sentences. Our results are consistent with Ahrens et al. (2007) who found that anomalous and familiar metaphors engaged right inferior frontal gyrus, and Bambini and colleagues who compared familiar and unfamiliar metaphors and found that both types of metaphors engaged bilateral inferior and right middle frontal gyri, anterior cingulate, right superior temporal gyrus, and left angular gyrus more than literal text (Bambini et al., 2011). Others have investigated the influence of novelty by using metaphoric and literal word pairs. Mashal and colleagues found greater engagement of right middle and inferior frontal gyri, and right superior temporal sulcus by novel metaphors compared with conventional metaphoric word pairs (Mashal et al., 2005, 2007).

Others have examined hemispheric recruitment using divided visual half field (VHF) paradigms in which processing can be initially biased toward one hemisphere by presentation to a single visual hemifield. Consistent with the role of the right hemisphere in processing novel metaphors, Faust and Mashal found that the left visual field/right hemisphere processed novel metaphoric pairs of words faster and more accurately compared to right visual field/left hemisphere presentation (Faust and Mashal, 2007; Mashal and Faust, 2009). Moreover, in a second study, Mashal and Faust showed that this right hemisphere advantage for novel metaphors disappeared with a second presentation of the word pairs, suggesting that as the stimuli became more familiar, the right hemisphere advantage disappeared (Mashal and Faust, 2009). However, others have failed to find a right hemisphere advantage for novel metaphoric pairs, instead finding comparable processing times for both novel literal and metaphoric word pairs and faster processing times for left hemisphere presentation for all stimuli (Forgács et al., 2014). Similarly, using electrophysiological recordings, Coulson and Van Petten (2007), found no laterality effects when examining low-cloze metaphorical sentences. However, they did find a greater N400 negativity to low-cloze metaphorical sentences compared with low-cloze literal sentences, consistent with an additional processing cost for metaphoric sentences even when they are equally predictable compared to literal sentences.

Collectively, these findings indicate that the right hemisphere could be sensitive to both novelty and figurativeness. While the novelty findings are consistent with all three theoretical frameworks, none of the frameworks would predict that familiar metaphors engage the right hemisphere. However, an alternative hypothesis is that even familiar metaphors present greater integration demands than familiar literal text in which case a parsimonious explanation would be that integration demands, rather than figurativeness *per se* are driving increased right hemisphere recruitment.

## Comprehension Difficulty Effects in Figurative Language

Comprehension difficulty may be another factor that contributes to the neural differences between figurative and literal language, however, few studies have explicitly manipulated this. Schmidt and Seger examined comprehension difficulty and familiarity in metaphoric sentences, including familiar and unfamiliar metaphors that were easy to understand and unfamiliar metaphors that were difficult to understand (Schmidt and Seger, 2009). In this case comprehension difficulty and familiarity were defined through a norming procedure conducted in a different set of participants. They found that all metaphoric sentences engaged right frontal regions and left temporal pole to a greater degree than literal sentences. Moreover, metaphors that were harder to understand engaged left inferior frontal gyrus more than metaphors that were easier to understand. These findings suggest that difficulty *per se*, does not explain the increased right hemisphere activation to metaphors.

## Context Effects in Figurative Language

Supportive context has repeatedly been shown to facilitate language processing from generating priming effects (Neely, 1991) to minimizing processing costs (Schwanenflugel et al., 1992). Context can be defined as the larger linguistic surroundings of a given word or phrase. It can consist of a single additional word, as in a prime-target pair or an entire sentence or paragraph. Moreover, some research has suggested that there may be hemispheric differences in context sensitivity with the right hemisphere processing primarily lexical level information and showing less sensitivity to sentential and discourse features (Faust et al., 1993, 1995, 2003). In contrast, other research has suggested that both right and left hemisphere processing of sentence-level information (e.g., Federmeier and Kutas, 1999a; St George et al., 1999; Federmeier, 2007). As we discussed previously, many figurative language experiments have used sentential stimuli, and found right hemisphere engagement (Bottini et al., 1994; Sotillo et al., 2005; Eviatar and Just, 2006; Stringaris et al., 2006; Ahrens et al., 2007; Chen et al., 2008; Schmidt and Seger, 2009; Yang et al., 2009;Diaz et al., 2011). Work from our lab that examined the role of context in processing sentential metaphors found that congruent, two-sentence texts engaged bilateral dorsal medial prefrontal cortex, right inferior frontal gyrus, and an anterior right temporal region (Diaz and Hogstrom, 2011). In other work examining the influence of discourse context, Prat and colleagues compared sentence passages in which the final, critical sentence was a metaphor (Prat et al., 2012). The preceding context could be neutral, or supporting a metaphoric, sarcastic, or literal interpretation. Although there were no effects of figurativeness *per se*, context effects were found in which the more difficult, sarcastic passages elicited greater activation than the passages supporting a metaphoric interpretation in bilateral dorsal medial frontal regions and bilateral inferior and middle frontal gyri. Additionally, Prat and colleagues found a negative correlation between readers’ vocabulary scores and activation in right inferior frontal gyrus, suggesting that individual differences may also influence right hemisphere activation in addition to contextual congruence.

However, of note, Bambini and colleagues examined metaphors in both a supportive and minimal context and did not find an effect of context on metaphor processing (Bambini et al., 2011). Additionally, other experiments have also failed to elicit right hemisphere activation despite using sentential figurative language (Rapp et al., 2004, 2007; Mashal et al., 2009). In these cases, it could be that the contexts did not vary in comprehension difficulty sufficiently.

## Conclusion

Numerous experimental studies have illustrated the important contributions of the right hemisphere to language comprehension, in particular semantic aspects. Although much of this work has highlighted enhanced recruitment of right hemisphere resources for figurative language, it is clear that many forms of language, including literal language also engage the right hemisphere. Here we examined three dimensions that influence comprehension and hemispheric recruitment (novelty, difficulty, and context). There are many other linguistic features and individual differences that also influence comprehension including aptness, valence, and theory of mind. Thus, increased right hemisphere activation likely reflects broader aspects of comprehension which we suggest are best encapsulated as integration difficulty.

## Author Contribuitions

MD conceived of the research topic. MD and AE reviewed the literature and wrote the manuscript.

## Conflict of Interest Statement

The authors declare that the research was conducted in the absence of any commercial or financial relationships that could be construed as a potential conflict of interest.
